# IL-10 and socs3 Are Predictive Biomarkers of Dengue Hemorrhagic Fever

**DOI:** 10.1155/2017/5197592

**Published:** 2017-07-30

**Authors:** Lilian Karem Flores-Mendoza, Tania Estrada-Jiménez, Virginia Sedeño-Monge, Margarita Moreno, María del Consuelo Manjarrez, Guadalupe González-Ochoa, Lourdes Millán-Pérez Peña, Julio Reyes-Leyva

**Affiliations:** ^1^Centro de Investigación Biomédica de Oriente, HGZ5, Instituto Mexicano del Seguro Social, Km 4.5 Carretera Atlixco-Metepec, 74360 Metepec, PUE, Mexico; ^2^Departamento de Ciencias Químico Biológicas y Agropecuarias, División de Ciencias e Ingeniería, Universidad de Sonora, 85880 Navojoa, SON, Mexico; ^3^Departamento de Ciencias de la Salud, Universidad Popular Autónoma del Estado de Puebla, 21 Sur 1103, 72410 Puebla, PUE, Mexico; ^4^Hospital General de Zona No. 5, Instituto Mexicano del Seguro Social, Km 4.5 Carretera Atlixco-Metepec, 74360 Metepec, PUE, Mexico; ^5^Laboratorio de Bioquímica y Biología Molecular, Centro de Química, Instituto de Ciencias, Benemérita Universidad Autónoma de Puebla, Edif. 103 F, CU-BUAP, San Manuel, 72570 Puebla, PUE, Mexico

## Abstract

**Background:**

Cytokines play important roles in the physiopathology of dengue infection; therefore, the suppressors of cytokine signaling (*socs*) that control the type and timing of cytokine functions could be involved in the origin of immune alterations in dengue.

**Objective:**

To explore the association of cytokine and *socs* levels with disease severity in dengue patients.

**Methods:**

Blood samples of 48 patients with confirmed dengue infection were analyzed. Amounts of interleukins IL-2, IL-4, IL-6, and IL-10, interferon- (IFN-) *γ*, and tumor necrosis factor- (TNF-) *α* were quantified by flow cytometry, and the relative expression of *socs1* and *socs3* mRNA was quantified by real-time RT-PCR.

**Results:**

Increased levels of IL-10 and socs3 and lower expression of *socs1* were found in patients with dengue hemorrhagic fever (DHF) with respect to those with dengue fever (DF) (*p* < 0.05). Negative correlations were found between *socs1* and both IL-10 and *socs3* (*p* < 0.01). The cutoff values of *socs3* (>199.8-fold), *socs1* (<1.94-fold), and IL-10 (>134 pg/ml) have the highest sensitivity and specificity to discriminate between DF and DHF.

**Conclusion:**

Simultaneous changes in IL-10 and *socs1/socs3* could be used as prognostic biomarkers of dengue severity.

## 1. Introduction

Dengue is one of the most important human viral diseases due to its large morbidity and economic impact [1, 2]. It is estimated that nearly half of the world's population lives in risk areas of dengue (3.6 billion). The disease causes over 230 million cases of dengue fever, more than 2 million cases of severe disease, and 21,000 deaths [1, 2]. In addition to the public health and economic cost, there is a major social impact in countries where large dengue epidemics occur, often disrupting primary care services due to the number of hospitalized patients during dengue outbreaks [1, 3]. Dengue is caused by a single-stranded RNA virus belonging to the Flaviviridae family and genus *Flavivirus*. Dengue virus (DENV) exists as four closely related serotypes.

Dengue symptoms include mild to high degree fever, retroorbital pain, headache, myalgia, arthralgia, rash, nausea, vomiting, abdominal pain, mucosal bleeding, and thrombocytopenia. Patients may deteriorate to severe dengue disease, including DHF and dengue shock syndrome (DSS), characterized by vascular permeability, plasma leakage, severe hemorrhage, and in some cases organ impairment and death [4–6].

The occurrence of DHF/DSS is thought to result from a complex interplay between virus and host immune factors [6]. Some studies found an increased risk of DHF after the second infection with a different serotype mediated by a process known as antibody-dependent enhancement (ADE) of infection and by cross-reactive autoantibodies and T cells [6–8].

Diverse efforts have been made to identify potential predictors of the development of severe disease (DHF/DSS). In those studies, several cytokines have been found elevated in patients with severe dengue compared to dengue without complications. But just a few of them, including IL-10, IL-6, and IFN-*γ*, have been proposed as potential predictors of disease severity [8–15].

Cytokines induce the activation of immune cells during infection, and their response is maintained under control by a group of negative regulatory factors, including the suppressors of cytokine signaling (SOCS), which are necessary to stop the cytokine cascade and return to homeostasis [16, 17]. Of the eight family members, *socs1* and *socs3* are the most potent inhibitors of cytokine signaling mediated by the JAK/STAT pathway [18, 19]. Overexpression of *socs1* and *socs3* is associated with deregulation of the immune response and the cytokine secretion patterns [16–19]. Several viruses destabilize the host antiviral defense by upregulating *socs1* and *socs3* [20–23].

The association between socs and dengue infection has been scarcely explored [24–29]. Ubol et al. and Tsai et al. reproduced the ADE phenomenon in cell lines infected with dengue virus in the presence of subneutralizing concentrations of antibodies. Under that condition, dengue virus infection induced more IL-10 and *socs3* expression, but reduced IFN-*β* production [24, 26]. Rolph et al. studied the ADE phenomenon in human primary macrophages, and they found that IL-6, but not IL-10, induced *socs3* expression and JAK-STAT pathway inhibition [25]. Chen et al. found high levels of IL-10 associated to reduce *socs1* expression in DHF patients [27]. We have previously found that DENV infection induced high expression of *socs1* and *socs3* in macrophages derived from the U-937 cell line, being *socs1* ten times more expressed than *socs3*. Those changes were associated with evasion of the antiviral innate immune response in dengue-infected cells [28, 29]. Here, we studied the immune response of patients during the first dengue outbreak identified in Puebla state, in a geographical area that delimits three dengue endemic states, but that differs in height above sea level and climate. We examined biochemical parameters, cytokines, and *socs* gene expression searching for correlations with dengue severity.

## 2. Methods

### 2.1. Ethical Statement

This research was approved by the Local Committee of Ethics and Research in Health of the Mexican Institute of Social Security (IMSS); registry number R-2011-2103-24. The study was performed in agreement with the Declaration of Helsinki (last actualization Brazil October 2013). Informed consent was obtained from patients soon after admission. In patients younger than 18 years, informed consent was obtained from their parents or legal custodian. Clinical data and blood samples were recorded with a serial study number to maintain confidentiality.

### 2.2. Patients' Data Collection and Laboratory Test

The study was done in patients admitted for medical attention for dengue at the General Hospital of Zone No. 5 (HGZ5), IMSS, located in Metepec, Puebla, Mexico, from August 1st to September 1st, 2013. The control group was integrated by healthy subjects from the blood bank of the Hospital of Specialties UMAE, IMSS, located in Puebla City, Mexico, which concentrates patients from a geographical region free of dengue.

Dengue diagnosis was confirmed by identifying the DENV-specific IgM and IgG (Mac-ELISA, PanBio Diagnostic), or the nonstructural protein 1 (NS1) antigen (Platelia Biorad).

As part of the medical attention, blood samples were taken for hematological and biochemical tests such as white blood cell counts, hematocrit, hemoglobin, platelet counts, albumin, aspartate (AST), and alanine (ALT) aminotransferases. Abdominal ultrasound and other tests were requested at a physician's discretion. Patients that completed the clinical history, physical examinations, and clinical laboratory tests and confirmed virus diagnosis were included in the immunological study group.

### 2.3. Case Classification

Cases were classified as DF and DHF according to WHO 1997 guidelines and hospital's internal directions. DF was defined as laboratory-confirmed cases with high fever without evidence of plasma leakage, with or without hemorrhagic manifestations. DHF was characterized by evidence of plasma leakage associated with the presence of hemorrhagic manifestations (petechia, ecchymosis, rash, or bleeding of the gastrointestinal mucosa, the urinary tract, or other locations) and thrombocytopenia (≤100,000 platelets/mm3) without shock.

### 2.4. Blood Sampling

For immunological purposes, whole blood samples were obtained either at 6 or 7 days of fever onset. Five mL of blood was drawn into heparinized tubes and centrifuged at 1700 rpm at 4°C for 7 minutes to separate plasma. Aliquots were stored at −70°C until use for flow cytometry. Peripheral blood mononuclear cells (PBMC) were isolated by Ficoll-Hystopaque density gradient centrifugation.

### 2.5. Flow Cytometry

Cytokine concentrations in plasma were measured with the BD cytometric bead array (CBA) Human Th1/Th2/Th17 Cytokine Kit (BD Biosciences), following the manufacturer's instructions. Samples were analyzed on the BD FACS Canto II Flow Cytometer and analyzed by FCAP ArrayTM Software (BD Bioscience). Cytokine standards were serially diluted, and calibration curves were constructed to determine the protein concentrations of the test samples.

### 2.6. Total RNA Extraction

Total ARN was isolated from PBMC using TRIzol (Invitrogen) according to the manufacturer's instructions. RNA was quantified using a plate reader spectrophotometer Synergy 4 (BioTek). RNA integrity was confirmed by agarose gel electrophoresis.

### 2.7. Quantitative Real-Time RT-PCR

Expression of *socs* genes was quantified by real-time RT-PCR. RNA was reverse transcribed using the random primers included in the RevertAid H Minus Reverse Transcriptase kit (Fermentas). The reaction mixture was incubated at 25°C for 10 min, 42°C for 60 min, and 70°C for 10 min. Real-time RT-PCR was performed using SYBR Green/ROX-PCR master mix (Fermentas). RNase P was used as an endogenous expression control in all experiments.

Dynamic range for *socs1* and *socs3* with respect to *RNase P* was calculated for both genes at 50–200 ng, giving a linear slope of 0.03 and 0.009, respectively. Assay specificity was confirmed by the dissociation curves corresponding to each gene.

The primers used were *socs1* Forward 5′-CAC GCA CTT CCG CAC ATT CC-3′, *socs1* Reverse 5′-TCC AGC AGC TCG AAG AGG CA-3′, *socs3* Forward 5′-ACA ATC TGC CTC AAT CAC TCT G 3′, and *socs3* Reverse 5′-TTG ACT TGG ATT GGG ATT TTG-3′.

All reactions were run in duplicate by using a StepOne Real-Time PCR system (Applied Biosystems). The mRNA expression level between T0 and Tn was expressed as an *n*-fold increase according to the formula to calculate relative expression 2^−ΔΔCT^ ± SD, where ∆CT is the difference in the threshold between any target gene (*socs1* or *socs3*) and the endogenous gene (*RNase P*) and ∆∆CT establishes the differences between study and control group conditions.

### 2.8. Statistical Analysis

Demographic and clinical characteristics were compared among subgroups by Mann–Whitney *U* test for continuous variables and *χ*^2^ test for categorical variables. The cytokine concentrations were expressed in pg/ml and were compared among groups using the Kruskal-Wallis followed by Dunn's posttest. Correlation coefficients were calculated with Pearson's test when the data had a normal distribution and with Spearman's when they had no normal distribution. A receiver-operating characteristic curve was made. Results were analyzed using GraphPRISM 5.0, and significant differences were considered at *P* < 0.05.

## 3. Results

### 3.1. Demographic Characteristics and Clinic Parameters

According to the hospital records, 73 patients were admitted due to dengue from August 1st to September 1st, 2013. Of these, only 48 patients fulfilled the requirements to be included in the study, accomplished all the clinical, virological, and immunological tests, and gave their informed consent. Based on the WHO classification of 1997, 38 patients presented DF and 10 DHF. Dengue infection was confirmed by measuring plasma levels of IgM in 37 patients, IgG in 7 patients, and NS1 in 4 patients. The age of the patients ranged from 12 to 62 years. The median age for DHF and DF patients was 41 and 31 years, respectively. The main characteristics of the study group are summarized in Table 1.

### 3.2. Clinical Signs

Most of the patients showed fever, headache, backache, arthralgia, and rash. Physical examinations did not reveal signs of plasma leakage such as ascites, pleural effusion, edema, or ecchymosis in any patient. Several DF patients presented some signs of bleeding: petechia (12/38), epistaxis (5/38), and gingival hemorrhage (3/38) were the most frequent. Most DHF patients presented two or more hemorrhagic signs: 4/10 presented petechiae in combination with epistaxis, hematomas, or gingival hemorrhage; and 3/10 presented a combination of petechiae, epistaxis, and gingival hemorrhage. Hematemesis and melena were less frequent (Table 1).

### 3.3. Hematological Analyses

Nonsignificant differences were observed between DF and DHF concerning the proportion of leukocytes, lymphocytes, monocytes, and neutrophils. Some parameters were significantly different between DHF and DF patients (Table 1), for example, the hematocrit (44.8 ± 5.4 versus 36.99 ± 9.94; *P* < 0.05), hemoglobin (14.94 ± 1.86 versus 12.21 ± 3.38; *P* < 0.05), and serum albumin levels (3.23 ± 0.58 versus 2.57 ± 0.65; *P* < 0.01). Platelet counts are important hallmarks of dengue disease, and <10.0 × 10^4^/mm3 values are indicative of high risk for hemorrhage. Low platelet counts were found in both DHF (3.9 ± 2.3 × 10^4^ cells/mm^3^) and DF patients (5.8 ± 7 × 10^4^ cells/mm^3^). Serum ALT levels were higher than the reference values (30–65 IU/ml for adults and not more than 48 IU/ml for children) at 65.73 ± 29.78 U/ml in DF patients and 138 ± 15.3 U/ml in DHF patients (*P* < 0.05).

### 3.4. Patterns of Serum Cytokines

**Figure 1:**
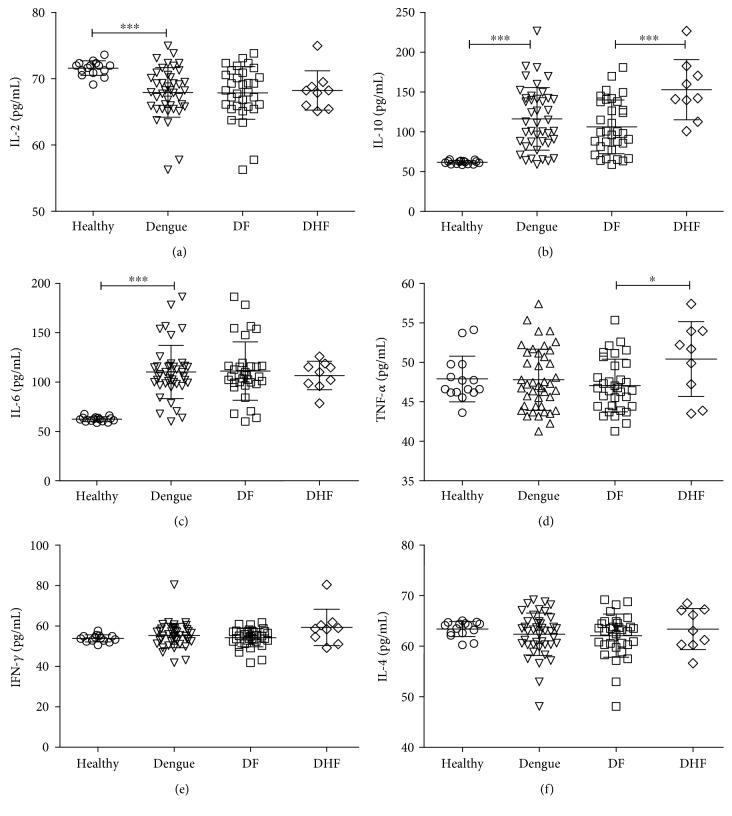
Cytokine concentrations in patients with dengue. Cytokines were determined by cytometric bead array in the blood plasma of the healthy subjects, in all patients with dengue, in DF, and DHF. Results show the values of all subjects belonging to each group; error bars represent mean ± S.D. Significant differences ^∗^*P* < 0.05; ^∗∗∗^*P* < 0.001 by Mann–Whitney *U* test for continuous variables.

Serum concentrations of IL-2, IL-4, IL-6, IFN-*γ*, TNF-*α*, IL-17, and IL-10 were determined in dengue patients at 6 or 7 days of fever onset and were compared with values obtained in a group of healthy controls. Dengue patients showed lower concentrations of IL-2 than controls (67.94 ± 3.75 pg/ml versus 71.59 ± 1.1 pg/ml; *P* < 0.001), but no significant differences in IL-2 between DF and DHF were found (67.86 ± 3.97 pg/ml versus 68.23 ± 2.96 pg/ml, resp.; Figure 1(a)).

IL-10 concentration was higher in dengue patients than in the control group (112.2 ± 26.97 pg/ml versus 59.58 ± 26.03 pg/ml, resp.; *P* < 0.001). In addition, the amount of IL-10 was notably higher in DHF than in DF (147.65 ± 36.48 pg/ml versus 102.58 ± 35.5 pg/ml, resp.; *P* < 0.001; Figure 1(b)).

A higher concentration of IL-6 was found in dengue patients compared to the control group (110.3 ± 37.88 pg/ml versus 62.48 ± 2.47 pg/ml; *P* < 0.001). However, there were no significant differences in IL-6 between DF and DHF patients (111.25 ± 29.58 pg/ml versus 106.7 ± 14.43 pg/ml; Figure 1(c)).

The mean plasma concentrations of TNF-*α* were 47.62 ± 2.83 pg/ml for the control group, 46.80 ± 3.26 pg/ml for DF, and 50.07 ± 4.63 pg/ml for DHF patients (*P* < 0.05; Figure 1(d)). There were no significant differences in IFN-*γ* or IL-4 values between the control group and dengue patients or between DF and DHF patients (Figures 1(e) and 1(f)). IL-17 was also evaluated, but the results were under the limit of detection of this assay (20 pg/ml).

### 3.5. Expression of *socs1* and *socs*3

**Figure 2:**
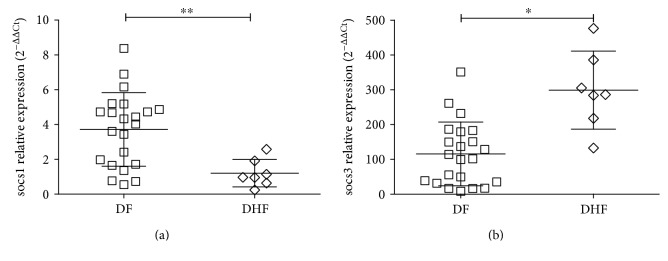
Relative expression of *socs* genes in patients with dengue. The relative expression of *socs1* and *socs3* mRNA was determined in PBMC of patients with DF and DHF. Results are expressed as fold increments compared with the expression level of the control group which was assigned an arbitrary value of 1. Error bars represent mean ± S.D. Significant differences ^∗^*P* < 0.05; ^∗∗^*P* < 0.01 by Mann–Whitney *U* test for continuous variables.

The relative expression of s*ocs1* and *socs3* genes in dengue patients was compared with their expression in the healthy control group, which was assigned an arbitrary value of 1. socs1 showed a significantly lower expression in DHF with respect to DF (1.24 ± 0.85-fold versus 4.34 ± 1.96-fold, resp.; *P* < 0.01; Figure 2(a)).

The relative expression of socs3 was higher than that of socs1 in all the patients. In addition, socs3 expression was directly proportional to disease severity. The relative expression of socs3 was 118.24 ± 94.57-fold for DF and 294.33 ± 110.67-fold for DHF (*P* < 0.0198; Figure 2(b)).

### 3.6. Correlation Analysis

**Figure 3:**
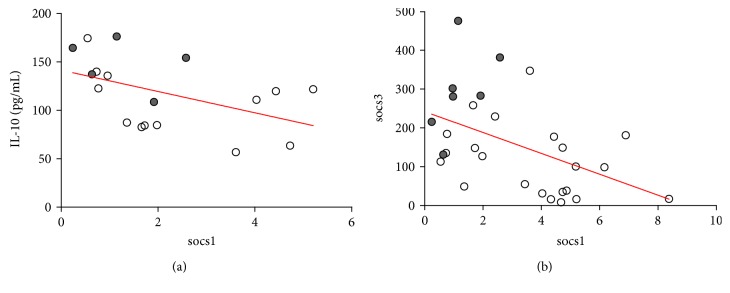
Correlation analysis between *socs1*, *socs3*, and IL-10. Spearman's and Pearson's correlation coefficients were calculated to identify the association between IL-10 concentration and socs1 expression (a) and between socs3 and socs1 expression levels (b).

Correlation coefficients were calculated between *socs1* and *socs3* expression levels, the concentrations of cytokines, and each biochemical data. Three correlations were statistically relevant: the negative correlation between IL-10 concentration and *socs1* (Spearman's *r* = −0.59, *P* < 0.01; Figure 3(a)), the negative correlation between *socs3* and *socs1* (Pearson's *r* = −0.48, *P* < 0.01; Figure 3(b)), and the positive correlation between IL-10 and lymphocyte counts (Spearman's *r* = 0.42, *P* < 0.05). All the other correlations were minimal (*r* < 0.4) or without significance.

### 3.7. Potential Biomarkers for Diagnosis and Prediction of Dengue Severity

The progress of dengue fever to its severe forms is one of the most concerning aspects of the disease, and there is no consensus biomarker for its prediction. As an approach, we employed an AUC (area under the curve) analysis to identify the potential of some parameters to discriminate between healthy and dengue-infected subjects, as well as between DF and DHF. IL-10, platelets, and IL-6 seemed to have a better diagnostic potential to discriminate between healthy and dengue patients, with 100% of specificity (SP) and 90.48%, 93.75%, and 95.24% of sensitivity (SE), respectively, for the cutoffs of >63 and >67 pg/ml for IL-10 and IL-6 and <13.6 × 10^4^ cell/mm^3^ for platelets (Table 2).

**Figure 4:**
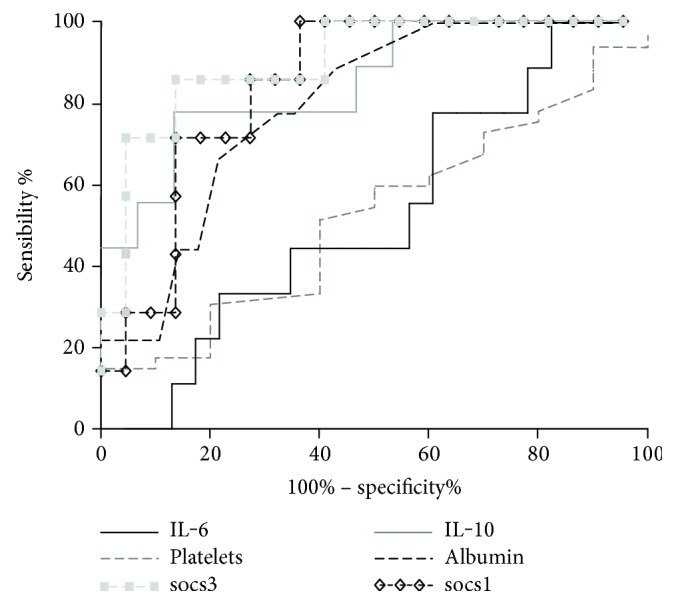
Predictive analysis of immunological biomarkers to identify dengue severity. The ROC curves were plotted using the sensitivity and specificity data and the cutoff values presented in Table 3, with the software GraphPad Prism 5. soc3, socs1, IL-10, and albumin discriminate between DF and DHF.

ALT, socs3, IL-10, and socs1 showed the best potential to predict dengue severity. ALT has 100% SE and 86% SP at a cutoff of >93.5 UI/ml. *socs3* has 85% SE and 86% SP at a cutoff of >199.8-fold. IL-10 has 77% SE and 86% SP at a cutoff of >134 pg/ml, and *socs1* has 85% SE and 72% SP at a cutoff of <1.948-fold (Table 3). The ROC curves were plotted, and their values indicate a good discrimination between DF and DHF (Figure 4). These analyses suggested that measuring these molecules in combination might contribute to predict disease severity. In this sense, the combination of IL-10 with socs1 and socs3 at the cutoffs mentioned might constitute a significant and biologically important biomarker of dengue severity at early stages of infection.

## 4. Discussion

Numerous factors are implicated in the progress of severe dengue disease such as viral load, serotype, and virulence, as well as genetic and immune host factors [8, 12, 30–34]. Several studies have evaluated the potential of clinical parameters in the prediction of severe dengue progress [35–38]. However, there is no consensus on the laboratory profiles that indicate dengue severity, given the significant variables introduced by local virus evolution, the intricacies of virus-host interactions, the geographical spread of the disease, the regional host genetics, and the stage of illness during sampling. Most research groups report the association of clinical manifestations with increased liver enzymes and albumin levels [38], as well as with leukocyte, hematocrit, and platelet counts [12, 36, 37]. In our study, increased liver enzymes and albumin levels correlated with severe dengue.

Several studies have associated the increased levels of cytokines with altered vascular permeability, plasma leakage, coagulopathy, and thrombocytopenia [6, 11, 14, 39–44]. However, there is still much controversy about the role of specific cytokines in determining the progress of DF to DHF, because the levels of these cytokines varied during the febrile and the critical phases [9, 14, 32, 39, 41, 45, 46]. TNF-*α* and IFN-*γ* are recognized as two of the most important factors associated to dengue progress [9, 34, 39]. IFN-*γ* seems to be relevant in the response to secondary infections [8, 34]. However, there were no differences in IFN-*γ* in our study, although we did not distinguish between primary and secondary infections. On the other hand, we found a slight increase in TNF-*α* in DHF patients. TNF-*α* has been found in a broad concentration range in dengue patients [34, 41, 42], that is, influenced by the time of blood sampling and TNF-*α*'s genetic polymorphisms [47, 48]. This variability makes TNF-*α* a nonideal marker for disease severity.

High concentrations of IL-6 were found in dengue patients in our study, and this is consistent with most studies [31, 42, 49–51]. IL-6 has been associated with the increment of vascular permeability, plasma leakage, pleural effusion, and ascites [41, 51, 52]. IL-6 induces leukocyte recruitment, local inflammation, and damage to endothelial cells. Thus, high levels of IL-6 correlate with a fatal outcome for DHF/DSS [43, 53].

Elevated IL-10 has been a consistent finding in patients with severe dengue. This led to propose IL-10 as a prognostic biomarker of DHF/DSS [11, 12, 26, 27, 34, 54]. The biological role of IL-10 in dengue pathogenesis is not fully known; it has been associated with changes in vascular permeability, plasma leakage, thrombocytopenia, and altered levels of hepatic transaminases [7, 8, 12, 40, 55–58]. However, the immunosuppressive function of IL-10 could be a major determinant of its contribution to dengue pathogenesis. IL-10 activates socs3 expression that inhibits the signaling pathways induced by IL-6, IL-2, IL-12, IFN-*γ*, and NF-kB. In turn, an increase in socs3 is associated with induction of Th2 cells and inhibition of the Th1 response [19, 55, 56]. Overexpression of IL-10 and *socs3* was associated with the ADE phenomenon in monocytes and macrophages infected with the dengue virus [24–26]. We found a high expression of IL-10 and socs3 in patients with severe dengue. Our results confirm in a larger population and a more quantitative way the results reported by Ubol et al. [24], supporting binomial IL-10/socs3 determination as a severe dengue biomarker.

socs1 is another negative regulator of cytokine signaling affected by dengue infection [28, 29]. Chen et al. reported a decrease in socs1 expression in DHF with respect to DF patients, which was associated with low levels of IFN-*γ* and high levels of IL-10 during defervescence [27], similar to our results. Low expression of socs1 seems to be associated to the ADE of infection phenomenon in cell cultures [24, 25, 35]. However, the presence of ADE is difficult to ascertain in patients.

Our results seem controversial because we found high levels of IL-6 and IL-10 in DHF patients. IL-10 is the major anti-inflammatory cytokine, and IL-6 is a potent proinflammatory factor. Both can block IFN-*α* activity by inducing socs3 [25]. socs3 is expressed by a feedback signal in response to IL-6, which led to a downregulation of IL-6 response and synthesis [44]. We have previously found that *socs3* overexpression in dengue-infected cells contributes to evasion of the innate immune response [28, 29]. In agreement, simultaneous increase of IL-6 and IL-10 in DHF is indicative of the dysregulated immune response that leads to the progress of dengue severity [49]. The changes in cytokine expression found in this study suggest that deregulation of *socs1* and *socs3* expression induced by dengue virus infection [28, 29] is not just involved in cytokine imbalance but also promotes the persistent inflammatory response that contributes to increased vascular permeability and hemorrhagic manifestations. However, further mechanistic studies are required to clarify how these regulatory factors are altered during dengue infection.

## 5. Conclusion

This study evidenced the association of *socs1* and *socs3* with the imbalance of cytokine response found in severe dengue patients. Our results suggest that the combined analysis of *socs1*, *socs3*, IL-10, and IL-6 could identify patients at risk of severe dengue.

## Figures and Tables

**Table 1:** Clinical characteristics of dengue patients during the outbreak of Puebla 2013.

	All dengue patients	DF	DHF	*P* values
	*N* = 48	*N* = 38	*N* = 10	
Gender (masculine/feminine)	24/24	21/17	3/7	
Mean age (range, years)	35.75 (12–59.5)	31 (12–58.5)	41 (12.5–62.5)	0.6938
*Laboratory data*				
Hematocrit (%)	43.16 ± 7.22	44.8 ± 5.4	36.99 ± 9.94	0.0351^∗^
Hemoglobin (g/dl)	14.37 ± 2.48	14.94 ± 1.86	12.21 ± 3.38	0.0223^∗^
Platelet count (×10^4^ cell/mm3)	5.41 ± 6.29^&^	5.8 ± 7	3.9 ± 2.3	0.7702
Albumin (U/ml)	3 ± 0.69^&^	3.23 ± 0.58	2.57 ± 0.65	0.0078^∗∗^
ALT (IU/ml)	80.05 ± 47.74^&^	65.73 ± 29.78	138 ± 15.3	0.0329^∗^
AST (IU/ml)	94.05 ± 59.75^&^	89.7 ± 54.77	165.6 ± 69.41	0.2179
White blood cells (×10^3^ cell/mm^3^)	5.46 ± 2.86	5.63 ± 3.09	4.83 ± 1.59	0.6221
Lymphocytes (%)	34.91 ± 13.89	33.44 ± 13.76	40.2 ± 13.77	0.3634
Monocytes (%)	11.96 ± 6.15	12 ± 6.42	11.8 ± 5.37	0.9150
Neutrophils (%)	52.37 ± 17.41	53.61 ± 17.53	47.9 ± 17.07	0.3049
*Clinical signs*				
Petechiae *n* (%)	19 (39.58)	12 (31.6)	7 (70)	0.0271^∗^
Epistaxis *n* (%)	10 (20.83)	5 (13.15)	5 (50)	0.0107^∗^
Gingival hemorrhage *n* (%)	7 (14.58)	3 (7.9)	4 (40)	0.0105^∗^
Hematoma *n* (%)	3 (6.25)	1 (2.63)	2 (20)	0.0435^∗^
Hematemesis *n* (%)	2 (4.16)	1 (2.63)	1 (10)	0.2995
Melena *n* (%)	2 (4.16)	1 (2.63)	1 (10)	0.2995

Data were obtained during the febrile period of the disease. Laboratory data express the average and standard deviation. Clinical data express the number of patients that presented the sign. *P* values were determined by Mann–Whitney *U* test for continuous variables and by the *χ*^2^ test for categorical variables. & means significance with respect to reference values. ∗, ∗∗ mean significance between DF and DHF.

**Table 2:** Potential biomarkers to discriminate healthy from dengue patients.

	AUC	95% CI	*P* value	Cutoff	Sensitivity %	Specificity %
IL-10	0.9683	0.9222–1.014	0.0001	>63.25 pg/ml	90.48	100
IL-6	0.9730	0.9311–1.015	0.0001	>67.77 pg/ml	95.24	100
Platelets	0.9742	0.9362–1.012	0.0001	<13.6 × 10^4^/mm^3^	93.75	100
Albumin	0.7594	0.5743–0.9245	0.0128	<3.55 UI/ml	78.38	63.64
ALT	0.9315	0.8263–1.037	0.0001	>42.5 UI/ml	83.33	100
AST	0.9167	0.8008–1.032	0.0001	>31.5 UI/ml	88.89	93.75

AUC analysis was done to identify parameters that can discriminate between healthy and sick dengue-infected patients. Sensitivity and specificity values are calculated on the base of the cutoff of each tested parameter.

**Table 3:** Potential biomarkers to identify severity of dengue infection.

	AUC	95% CI	*P* value	Cutoff	Sensitivity %	Specificity %
IL-10	0.8519	0.6922–1.011	0.0046	>134 pg/ml	77.78	86.67
IL-6	0.5266	0.3120–0.7412	0.8177	<136.8 pg/ml	100	17.39
Platelets	0.5316	0.3400–0.7231	0.7607	<9.05 × 10^4^/ml	100	15.79
Albumin	0.7976	0.6942–0.9461	0.0079	<3.55 UI/ml	88.89	57.14
ALT	0.9111	0.7682–1.054	0.0284	>93.5 UI/ml	100	86.67
AST	0.8000	0.5367–1.063	0.1098	> 140 UI/ml	86.67	66.67
socs1	0.8441	0.7006–0.9877	0.0069	< 1.94-fold	85.71	72.73
socs3	0.9026	0.7777–1.028	0.0016	>199.8-fold	85.71	86.36

AUC analysis was done to explore the potential of each parameter to predict dengue severity by comparing between DF and DHF patients. socs3, socs1, IL-10, and ALT have the best predictive values.

## Abbreviations

DENV: Dengue virus
PBMC: Peripheral blood mononuclear cell
SOCS: Suppressor of cytokine signaling
NS1: Nonstructural protein 1
TNF-*α*: Tumor necrosis factor alpha
IFN-*α*: Interferon-alpha
IFN-*γ*: Interferon-gamma
RT-PCR: Reverse transcription-polymerase chain reaction
DF: Dengue fever
DHF: Dengue hemorrhagic fever
DSS: Dengue shock syndrome
MIP-1: Macrophage inflammatory protein-1
STAT: Signal transducers and activators of transcription
JAK: Janus kinase
NF-kB: Nuclear factor kB
FBS: Fetal bovine serum
CT: Cycle threshold.
